# Machine learning models to predict systemic inflammatory response syndrome after percutaneous nephrolithotomy

**DOI:** 10.1186/s12894-024-01529-1

**Published:** 2024-07-08

**Authors:** Tianwei Zhang, Ling Zhu, Xinning Wang, Xiaofei Zhang, Zijie Wang, Shang Xu, Wei Jiao

**Affiliations:** 1 Department of Urology, The Affiliated Hospital of Qingdao University, Qingdao, China; 2 Shandong Key Laboratory of Digital Medicine and Computer Assisted Surgery, The Affiliated Hospital of Qingdao University, Qingdao, China; 3 Department of Education and Training, The Affiliated Hospital of Qingdao University, Qingdao, China

**Keywords:** Machine learning, Percutaneous nephrolithotomy, Relevant factors, Systemic inflammatory response syndrome

## Abstract

**Objective:**

The objective of this study was to develop and evaluate the performance of machine learning models for predicting the possibility of systemic inflammatory response syndrome (SIRS) following percutaneous nephrolithotomy (PCNL).

**Methods:**

We retrospectively reviewed the clinical data of 337 patients who received PCNL between May 2020 and June 2022. In our study, 80% of the data were used as the training set, and the remaining data were used as the testing set. Separate prediction models based on the six machine learning algorithms were created using the training set. The predictive performance of each machine learning model was determined by the area under the receiver operating characteristic curve (AUC), accuracy, sensitivity and specificity using the testing set. We used coefficients to interpret the contribution of each variable to the predictive performance.

**Results:**

Among the six machine learning algorithms, the support vector machine (SVM) delivered the best performance with accuracy of 0.868, AUC of 0.942 (95% CI 0.890–0.994) in the testing set. Further analysis using the SVM model showed that prealbumin contributed the most to the prediction of the outcome, followed by preoperative urine culture, systemic immune-inflammation (SII), neutrophil to lymphocyte ratio (NLR), staghorn stones, fibrinogen, operation time, preoperative urine white blood cell (WBC), preoperative urea nitrogen, hydronephrosis, stone burden, sex and preoperative lymphocyte count.

**Conclusion:**

Machine learning-based prediction models can accurately predict the possibility of SIRS after PCNL in advance by learning patient clinical data, and should be used to guide surgeons in clinical decision-making.

## Introduction

Kidney stones are one of the most common urological diseases, and its prevalence is reported to be increasing worldwide [1]. According to reports, Chinese adults have a 5.8% incidence rate of renal calculi, with approximately 1 in 17 adults currently diagnosed [1, 2]. Since the first report on percutaneous nephrolithotomy (PCNL) in 1976, it has gradually become the standard of care for patients with calculi larger than 2 cm, multiple or staghorn [3].

PCNL has certain advantages including minimal trauma, high stone clearance rates, short hospital stays and quick recovery. However, it also has several complications after surgery. Systemic inflammatory response syndrome (SIRS) is a common and serious complication associated with PCNL, with an incidence rate of 16.7-27.4% [4]. A urosepsis incidence between 0.3% and 4.7% can develop from postoperative SIRS if not diagnosed and treated early [5]. When sepsis progresses to septic shock or multiple organ failure, it causes high mortality rates and increased treatment costs.

Machine learning algorithms have increasingly been used to aid diagnosis, treatment, and automatic classification in medicine as statistical theory and computer technology have developed [6]. In the past studies, machine learning models has been uesd to predict the acute kidney injury after nephrectomy in patients with renal cell carcinoma and showed good predictive performance [7]. Machine learning algorithms was used to develop to predict the risk of incontinence after robot-assisted radical prostatectomy [8]. Due to the fact that machine learning algorithms show great potential in processing complex data sets, we should develop an efficient prediction model based on machine learning to identify patients with potential risk factors for post-PCNL SIRS and closely monitor their vital signs after surgery, which can significantly reduce the burden of false alarms. The main objective of this study was to analyze the influencing factors of SIRS after PCNL. Multiple machine learning algorithms were used to construct and verify the prediction model of SIRS after surgery. The performance of each machine learning model is compared, and the optimal prediction model is proposed.

## Materials and methods

### Patients

**Fig. 1:**
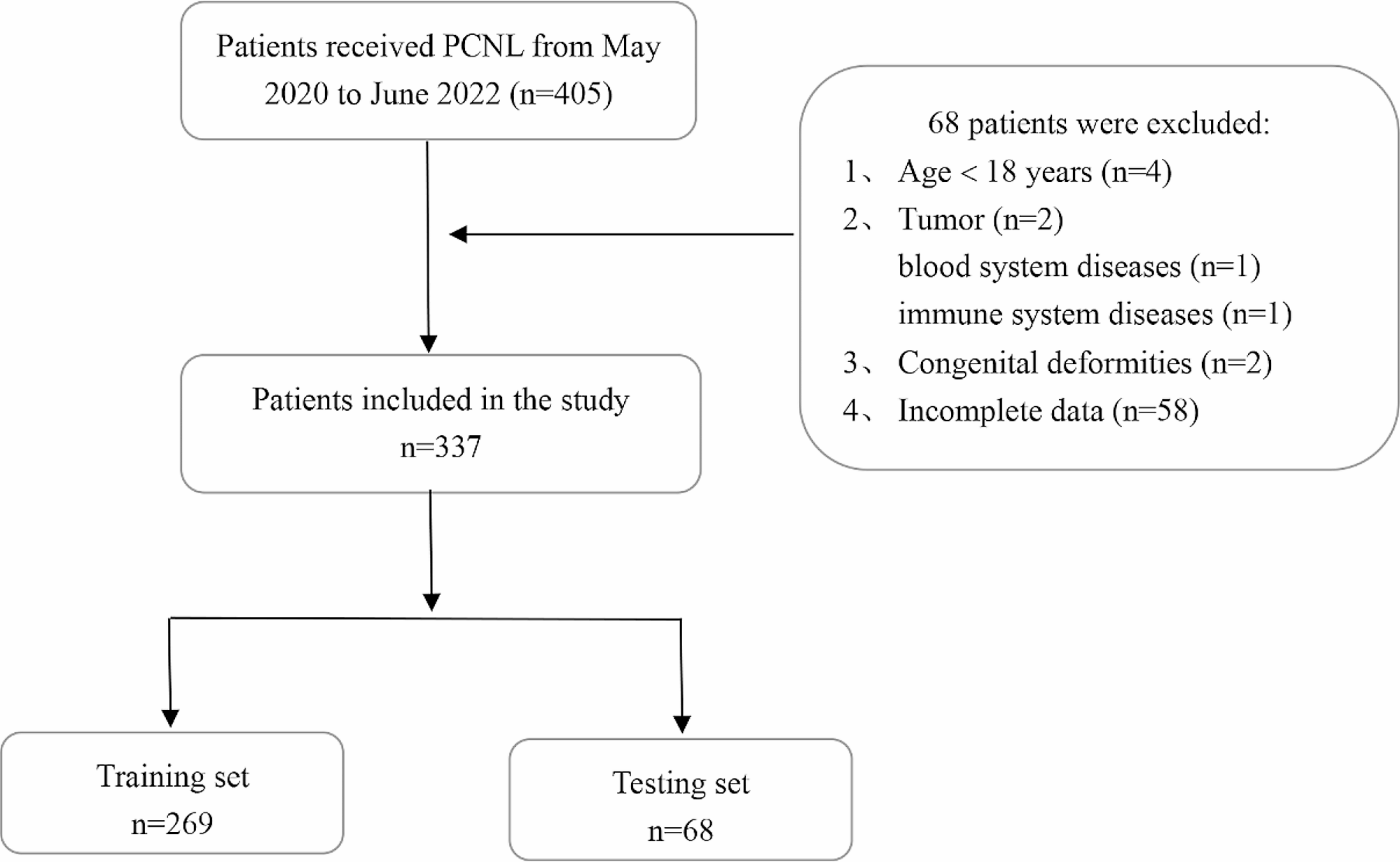
Flow chart illustrating the process of patient enrollment

We retrospectively collected the clinical data of 337 patients who underwent PCNL in the urology department of the affiliated hospital of Qingdao University between May 2020 and June 2022 by a single surgeon. Patients were excluded from the analysis based on the following criteria: (a) a history of bilateral PCNL; (b) the presence of patients with tumors, blood system or immune system diseases; (c) congenital deformities such as polycystic kidney, horseshoe kidney and solitary kidney; and (d) missing data. This study complied with the principles of the Declaration of Helsinki and was conducted in accordance with the ethical standards of the medical ethics committee of our institution. The patient selection flow diagram was shown in Fig. 1.

### Data collection

The preoperative data on the patients included: age, sex, body mass index (BMI), preoperative white blood cell (WBC), neutrophil (N), lymphocyte (L), monocyte (M), platelet (PLT), hemoglobin (HB), neutrophil to lymphocyte ratio (NLR), platelet to lymphocyte ratio (PLR), lymphocyte to monocyte ratio (LMR), systemic immune-inflammation (SII, neutrophil count × platelet countn / lymphocyte count), preoperative serum creatinine, urea nitrogen, uric acid, cystatin, albumin, prealbumin, fibrinogen, stone burden (length × width × π × 0.25), urine WBC, urine nitrite, urine culture, and hydronephrosis. Intraoperative information included operation time. Postoperative information uniformly measured at 6 am on the first day after surgery included peripheral WBC count, blood pressure, heart rate, oxygenation, respiratory rate, and body temperature.

Patients with SIRS were diagnosed if they met two or more of the following four criteria: (a) leukocyte count < 4 × 10^9^ cells/L or > 12 × 10^9^ cells/L; (b) body temperature > 38 °C or < 36 °C; (c) heart rate > 90/min; and (d) respiratory rate > 20/min or PaCO2 < 32 mmHg [9].

### Feature selection

Firstly, we conducted the statistical t-test, Mann–Whitney test and chi-square test for clinical characteristics to determine whether there are differences between the groups. Our analysis considered the feature significant when its two-tailed *p*-value was *p* < 0.05. Secondly, spearman correlation analysis was performed to reduce collinearity among features. To reduce the risk of overfitting, the least absolute shrinkage and selection operator (LASSO) algorithm was applied to select features with non-zero coefficient values.

### Model building

To predict the occurrence of post-PCNL SIRS, we used six machine learning algorithms, including the support vector machine (SVM), light gradient boosting machine (LightGBM), eXtreme gradient boosting (XGBoost), k-nearest neighbor (KNN), random forest (RF) and Extra-Trees classifier. These algorithms cover a wide range of modeling methods, ensuring that we can capture complex patterns in our data and improve the accuracy of our predictions. SVM is suitable for high-dimensional data and small samples, and has good classification effect. LightGBM and XGBoost are integrated methods based on decision tree, which have excellent performance and fast training speed when dealing with complex data sets. As a simple nonparametric method, KNN is easy to understand and apply. Random forest and Extra-Trees enhance the robustness and stability of the model through the integration of multiple decision trees. Choosing these algorithms allows us to comprehensively evaluate the performance of different models in predicting SIRS, ensuring that we get the best prediction results.

The data were randomly categorized into training set (80%) and testing set (20%). The training set includes 216 patients without SIRS and 53 patients with SIRS, while the testing set includes 54 patients without SIRS and 14 patients with SIRS. The training set was used to establish the prediction models using five-fold cross-validation, whereas the testing set was used to validate the prediction models using the area under the curve (AUC) of the receiver operating characteristics (ROC). We calculated the correlation coefficient between features which was used to visualize the contribution of each feature to the model predictions.

### Statistical analysis

Continuous variables with normal distributions were presented as mean ± standard deviation (SD) and compared using Student’s t-test. Continuous variables with non-normal distributions were presented as medians with interquartile ranges and compared using the Mann–Whitney test. Categorical variables were expressed as frequencies with proportions and compared using the chi-square test. The machine learning models were written in Python 3.7 language. LASSO algorithm and correlation analysis were implemented by importing the “scipy”, “numpy”, and “sklearn” packages in Python (version 3.7), and were performed using the “One-key AI” platform (http://www.medai.icu/), which was based on Python (version 3.7). The code used in this study was derived from: https://gitee.com/wangqingbaidu/OnekeyCompo. The area under the curve (AUC) was used to evaluate the predictive effectiveness of models, and DeLong test was used to compare whether the efficiency differences between the models were statistically significant. A bilateral *P*-value < 0.05 was considered as a measure of statistical significance.

## Results

### Patient characteristics

The study included 337 patients who underwent PCNL and had complete medical records. Among these, 69 patients experienced SIRS. The patients were divided into two groups based on whether SIRS occurred after PCNL. The age, sex, preoperative N, preoperative L, preoperative PLT, preoperative Hb, uric acid, serum albumin, serum fibrinogen, serum prealbumin, NLR, PLR, LMR, SII, operation time, stone burden, urine WBC, urine culture, and staghorn stones were significantly different between the two groups. The baseline data of the included patient are shown in Table 1.

**Table 1:** Comparison of clinical factors between patients with and without postoperative SIRS

Variable name	No SIRS	SIRS	*P* value
Age (years)	55.63 ± 10.69	58.09 ± 10.62	0.017
BMI (kg/m^2^)	25.56 ± 3.51	24.88 ± 3.20	0.135
Preoperative WBC (109/L)	6.67 (5.48,7.54)	6.94 (5.45,7.71)	0.396
Preoperative N (109/L)	3.75 (3.03,4.50)	4.34 (3.29,5.27)	0.004
Preoperative L (109/L)	2.08 (1.64,2.44)	1.52 (1.80,2.10)	<0.001
Preoperative M (109/L)	0.47 (0.40,0.56)	0.42 (0.33,0.59)	0.042
Preoperative PLT (109/L)	237.50 (195.75,275.00)	262.80 (218.00,308.00)	0.005
Preoperative HB (g/L)	143.15 ± 19.70	136.10 ± 33.83	<0.001
Preoperative serum creatinine (umol/L)	69.00 (56.00,84.00)	72.00 (59.00,85.00)	0.569
Preoperative urea nitrogen (mmol/L)	6.26 (5.33,7.55)	6.79 (5.52,8.13)	0.039
Preoperative cystatin (mg/L)	0.96 (0.82,1.17)	0.99 (0.84,1.18)	0.392
Preoperative uric acid (umol/L)	359.00 (292.75, 431.25)	326.00 (263.00,398.00)	0.010
Serum albumin (g/L)	44.29 ± 4.19	43.21 ± 4.79	0.2
Serum fibrinogen (g/L)	3.20 ± 0.64	3.64 ± 0.75	0.001
Serum prealbumin (mg/L)	337.20 ± 68.64	295.20 ± 66.17	0.001
NLR	1.88 (1.44,2.32)	2.41 (1.85,2.92)	<0.001
PLR	116.10 (92.25,431.25)	147.24 (112.36,176.19)	<0.001
LMR	4.56 (3.61,5.68)	4.14 (3.08,5.07)	0.018
SII	440.42 (327.51,555.50)	632.34 (456.18,807.04)	<0.001
Operation time (min)	80.00 (65.00,100.25)	95.00 (75.00,125.00)	<0.001
Stone burden (mm^2^)	365.20 (224.62,837.21)	676.21 (307.87,1425.46)	0.002
Gender (male/female); N (%)			
Male	179 (66.30)	34 (50.75)	0.018
Female	91 (33.70)	33 (49.25)	
Preoperative urine nitrite; N (%)			
No	192 (71.11)	43 (64.18)	0.269
Yes	78 (28.89)	24 (35.82)	
Preoperative urine WBC; N(%)			
No	65 (24.07)	7 (10.45)	0.017
Yes	205 (75.93)	60 (89.55)	
Staghorn stones; N (%)			
No	135 (50.00)	17 (25.37)	<0.001
Yes	135 (50.00)	50 (74.63)	
Preoperative urine culture; N (%)			
No	196 (72.59)	27 (40.30)	<0.001
Yes	74 (27.41)	40 (59.70)	
Hydronephrosis; N (%)			
No	221 (81.85)	36 (53.73)	0.001
Yes	49 (18.15)	31 (46.27)	

BMI body mass index, WBC white blood cell, N neutrophil, L lymphocyte, M monocyte, PLT platelet, HB hemoglobin, NLR neutrophil to lymphocyte ratio, PLR platelet to lymphocyte ratio, LMR Lymphocyte to monocyte ratio, SII Systemic immune-inflammation

### Feature selection and model building

**Fig. 2:**
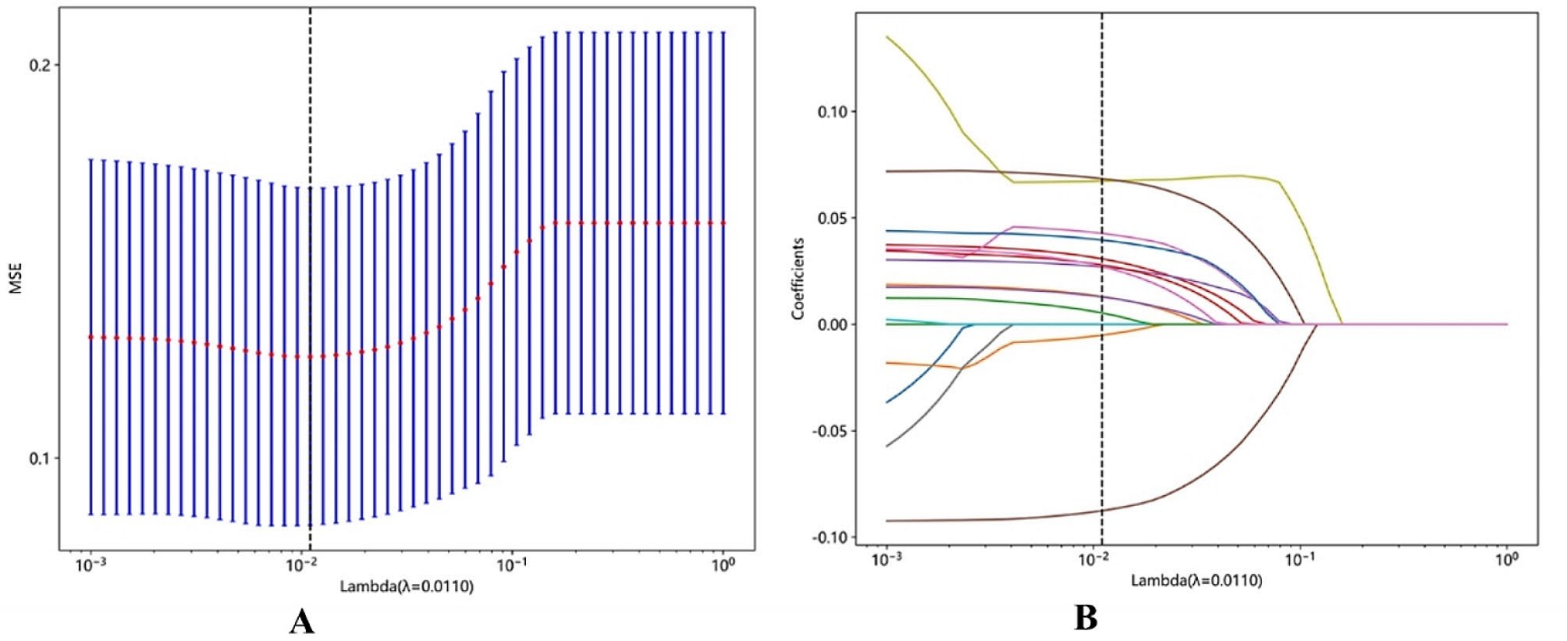
**A** The process of feature selection. We used the least absolute shrinkage and selection operator (LASSO) regression model with penalty parameter tuning conducted by fivefold cross validation according to minimum criteria. Selection of the tuning parameter (λ). Based on the minimum criteria, the vertical dotted line is plotted at the optimal value λ = 0.0110. **B** The vertical line was plotted with 13 selected features

Using spearman correlation analysis and the lasso algorithm with fivefold cross-validation (Fig. 2A, B), the 27 variables were ultimately reduced to 13 potential predictors of post-PCNL SIRS risk (Table 2), which were incorporated into the construction of the predictive model in our study.

**Table 2:** Feature selection results and coeffients for each feature

Variable name	Coefficient
Serum prealbumin	-0.076555
Preoperative urine culture	0.069698
Preoperative SII	0.057778
Preoperative NLR	0.045636
Staghorn stones	0.037962
Serum fibrinogen	0.034826
Operation time	0.028595
Preoperative urine WBC	0.026921
Preoperative urea nitrogen	0.021396
Hydronephrosis	0.017451
Stone burden	0.013898
Sex	0.007524
Preoperative L	-0.000457

SII Systemic immune-inflammation, NLR neutrophil to lymphocyte ratio, WBC white blood cell, L lymphocyte

**Fig. 3:**
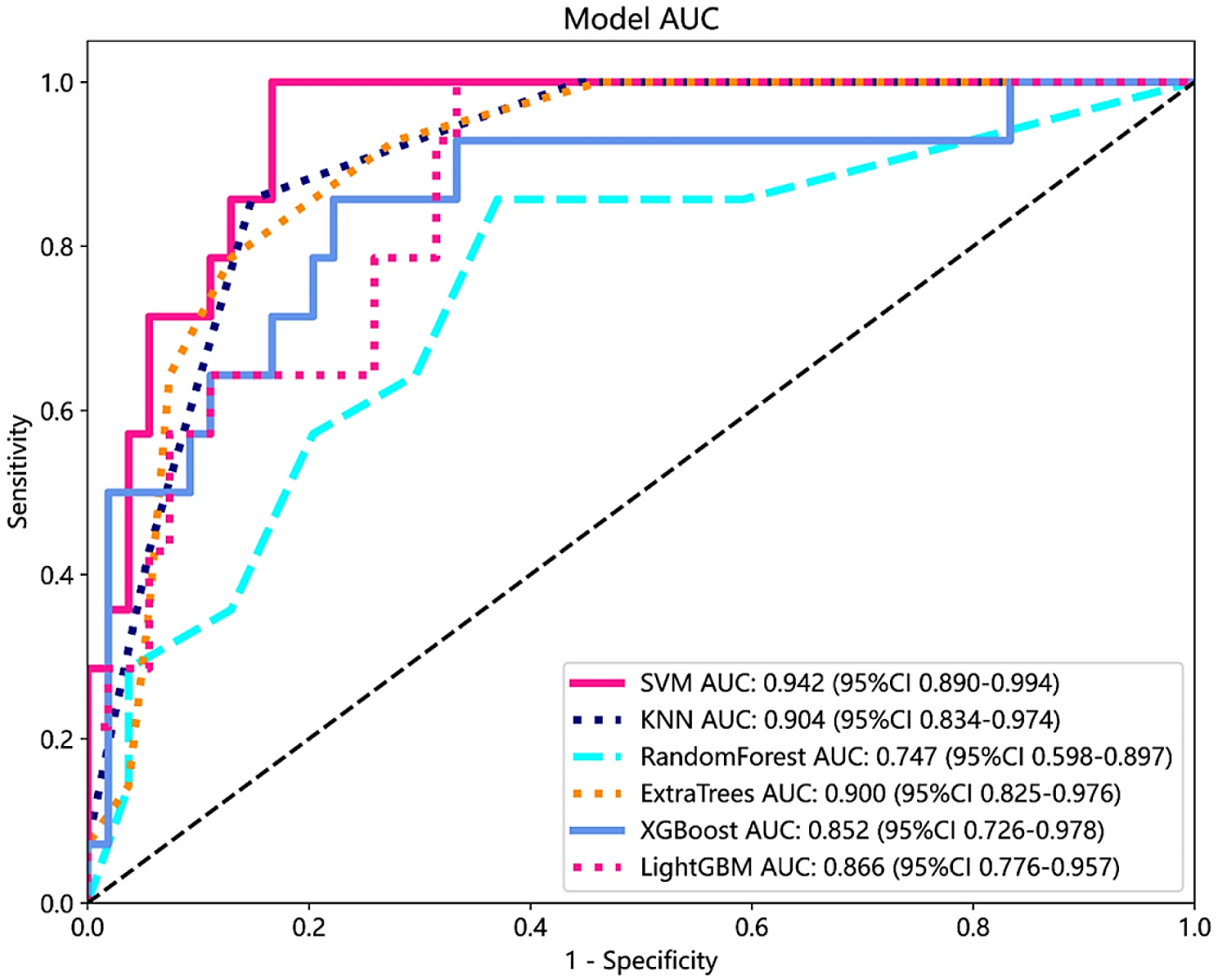
Performance for machine learning models based on the AUC of the ROC curve. The SVM model performed the best predictive ability. AUC area under the curve, ROC receiver operating characteristic, SVM support vector machine, KNN k-nearest neighbor, LightGBM light gradient boosting machine, XGBoost eXtreme gradient boosting

The training set and testing set consisted of 80% and 20% databases, respectively. Six machine learning algorithms were used to establish the prediction models in the training set, and the performance of the models was evaluated using the testing set and expressed by the AUC, accuracy, sensitivity, and specificity. The six machine learning algorithms that were used to predict SIRS following PCNL used the 13 selected factors as inputs. The performance results of the prediction models are shown in Table 3. The receiver operating characteristics (ROC) and the area under the curve (AUC) for each different prediction models were shown in Fig. 3. The results revealed that the SVM model (AUC=0.942) outperformed KNN model (AUC = 0.904, *P* = 0.046), Extra-Trees model (AUC = 0.900, *P* = 0.038), LightGBM (AUC = 0.866, *P* = 0.014), XGBoost (AUC = 0.852, *P*<0.001), and Random Forest (AUC = 0.747, *P*<0.001). The SVM model performed the best predictive ability than other models to predict the occurrence of SIRS after PCNL.

**Table 3:** Comparison of the performance of machine learning models in the training and testing set

Set	Model	Accuracy	AUC (95% CI)	Sensitivity	Specificity
Training set	Support Vector Machine	0.885	0.948 (0.913,0.983)	0.925	0.875
	LightGBM	0.829	0.952 (0.924,0.980)	0.981	0.792
	Extra-Trees	1.000	1.000 (1.000,1.000)	1.000	1.000
	XGBoost	0.981	0.998 (0.995,1.000)	0.981	0.981
	Random Forest	0.978	0.999 (0.997,1.000)	0.981	0.977
	K-Nearest Neighbor	0.870	0.927 (0.897,0.958)	0.811	0.888
Testing set	Support Vector Machine	0.868	0.942 (0.890,0.994)	1.000	0.833
	LightGBM	0.735	0.866 (0.776,0.957)	1.000	0.667
	Extra-Trees	0.853	0.900 (0.825,0.976)	0.786	0.870
	XGBoost	0.794	0.852 (0.726,0.978)	0.857	0.778
	Random Forest	0.676	0.747 (0.598,0.897)	0.857	0.642
	K-Nearest Neighbor	0.853	0.904 (0.834,0.974)	0.857	0.852

LightGBM, light gradient boosting machine; XGBoost, eXtreme gradient boosting

**Fig. 4:**
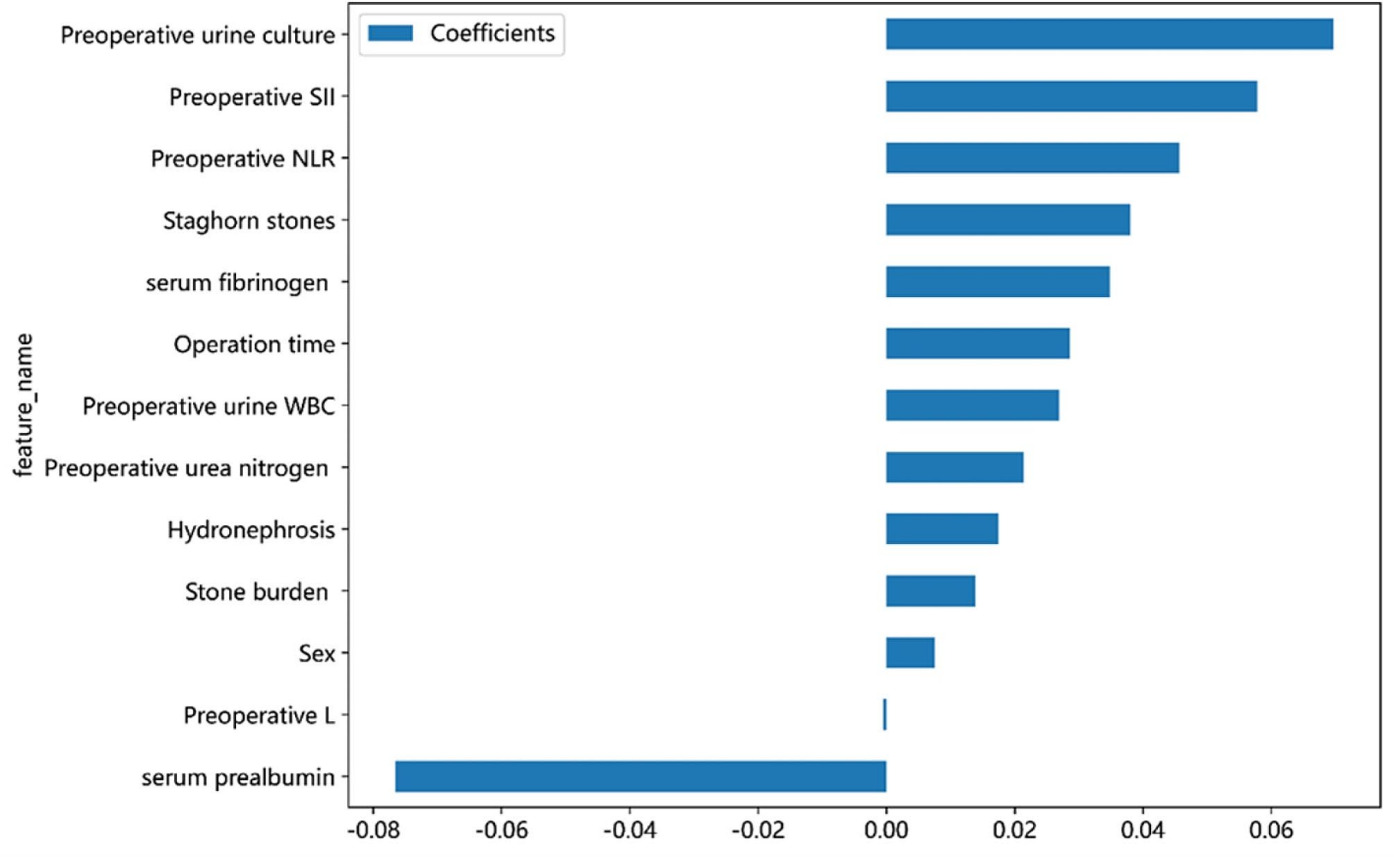
Top 13 selected features and the corresponding variable coefficients. Y-axis shows the top 13 variables, X-axis shows their impact on the machine model. L lymphocyte, SII Systemic immune-inflammation, NLR Neutrophil to lymphocyte ratio

Coefficients were used to interpret the results of the best prediction model by evaluating the contribution of each variable to the prediction model in Fig. 4. As shown in Fig. 4, further analysis revealed that prealbumin contributed the most to the prediction of the outcome, followed by preoperative urine culture, systemic immune-inflammation (SII), neutrophil to lymphocyte ratio (NLR), staghorn stones, fibrinogen, operation time, preoperative urine white blood cell (WBC), preoperative urea nitrogen, hydronephrosis, stone burden, sex and preoperative lymphocyte count.

## Discussion

PCNL has become the first choice of treatment for multiple or staghorn renal calculi larger than 2 cm [10]. Although PCNL has the advantage of less trauma and higher stone removal rate, PCNL still has many complications compared with other minimally invasive stone surgical techniques, particularly postoperative bleeding and postoperative infection. Typical clinical symptoms are insufficient during the initial stages of SIRS, making it difficult to detect post-PCNL SIRS at an early stage. SIRS may cause sepsis or multiple organ failure if untreated promptly. Therefore, we should develop a suitable prediction model based on machine learning algorithms for early detection of SIRS.

With the evolution of statistical theory and computer technology, novel machine learning techniques have improved predictive performance compared with traditional prediction methods. Previous studies have shown that machine learning algorithms can be utilized to predict the occurrence of SIRS in intensive care unit (ICU) or emergency department (ED) patients [11]. Kijpaisalratana et al. developed prediction models for early sepsis in the ED [12]. Hou et al. developed a model for predicting mortality in intensive care units for patients with sepsis using the XGboost algorithm [13]. However, few studies have discussed machine learning-based models for predicting SIRS after PCNL.

To our knowledge, this is the first time that machine learning algorithms have been used to predict post-PCNL SIRS. In this study, we compared the performances of six machine learning algorithms at predicting SIRS after PCNL. We take the following steps to address the potential bias inherent in retrospective studies and single-center data collection: Firstly, our rigorous data collection: ensures data consistency and accuracy to reduce information bias; Secondly, potential confounding factors were controlled through multivariate analysis to improve the reliability of the results, thereby reducing the bias impact of single-center data collection.

We mitigate the risk of model overfitting by the following measures: Firstly, we use the LASSO algorithm for feature selection, which can reduce the model complexity and prevent overfitting by increasing regularization terms. Secondly, the 5-fold cross-validation method is used to randomly divide the dataset several times during the training process and evaluate the model performance. Cross-validation can effectively evaluate the generalization ability of the model and prevent overfitting. Finally, we build the model through a variety of machine learning algorithms (such as support vector machine, K-nearest neighbor, random forest, eXtreme Gradient Boosting, Extra-Trees, LightGBM). By comparing the performance of different models, the model with the best performance can be selected, thus improving the model generalization ability.

In our study, the SVM model outperformed the other prediction models in terms of AUC. The excellent performance of the SVM model is mainly attributed to their good adaptability to small sample and high-dimensional datasets, as well as the effectiveness of their kernel trick in dealing with nonlinear problems. This is particularly important for common data characteristics in the medical field. In addition, the SVM model reduces overfitting by regularizing parameters, ensuring the generalization ability of the model. Therefore, we believe that SVM is a reasonable choice for this study.

We used the weight coefficient to quantify each variable’s contribution to the SVM model. Preoperative urine culture, SII, NLR, staghorn stones, fibrinogen, operation time, urea nitrogen, hydronephrosis, preoperative urine WBC, stone burden, and gender were potential risk factors. Previous studies have shown that the SII was a promising prognostic indicator in hepatocellular carcinoma [14], colorectal cancer [15], gastric cancer [16], prostate cancer [17] and post-PCNL SIRS [18]. Stones released inflammatory mediators, such as IL-6, IL-7, IL-8, and TNF-α, as well as increase in neutrophils [19]. Platelets were rich in pro-inflammatory factors and can release active inflammatory metabolites [20]. Excessive inflammatory reactions inhibited the immune responses and reduced the number of lymphocytes, which was associated with an increase in the SII value. SII, an inexpensive and readily available biomarker that can comprehensively reflect the immune status of the host, contributed the most to outcome prediction in our study. In this study, the contribution of NLR to the output of the SVM model was the second most accurate predictor of blood routine after SII. According to Kriplani et al., the NLR was an easily accessible and cost-effective predictor of post-PCNL SIRS [21]. As shown in Fig. 4, preoperative lymphocytes served as a protective factor. Jager et al. highlighted that lymphocytopenia was a sign of bacteremia because of accelerated apoptosis [22]. Therefore, patients with higher NLR, SII, or lymphocytopenia should receive more consideration.

Similar to previous studies, we found that positive urine culture, positive urine WBC, staghorn stone, stone burden and operation time were key factors associated with SIRS after PCNL [23]. Although recent research have shown that renal pelvic urine culture and stone culture can more accurately predict urosepsis than mid-section urine culture, it took a long time to cultivate renal pelvic urine and calculi [24]. Therefore, mid-bladder urine culture remained a reliable indicator because it can be easily obtained. Positive urine WBC indicated a urinary infection, which can increase the risk of SIRS after PCNL [25]. Therefore, it is necessary to complete routine urine tests and urine culture examinations before PCNL to evaluate the severity of urinary tract infection. Patients with urinary tract infections. need to receive adequate anti-infection treatment before surgery. The operation cannot be performed until the infection is controlled.

It has been reported that staghorn stones may be associated with postoperative sepsis because they harbored colonized bacteria, making it difficult to sterilize urine prior to surgery [26]. A larger stone burden made the operation more difficult and prolonged the operation time, which may prolong renal pelvis pressure and increase the risk of endotoxin absorption into the blood [18]. Thus, more attention should be paid to patients with staghorn stones, larger stone burden, or prolonged operative time.

Several literature reports have described that fibrinogen and prealbumin are related factors for postoperative SIRS [21]. Fibrinogen was a key regulator of inflammation in lung cancer [27], hepatocellular carcinoma [28], colorectal cancer [29] and urological cancers [30]. The prealbumin level was a protective predictor in Fig. 4. The prealbumin level reflected the recent nutritional status of a patient and had a positive effect on the prognosis of disease. Therefore, more attention should be paid to high-risk patients with high fibrinogen and low prealbumin levels.

Gender was an independent risk factor for post-PCNL SIRS. This was related to the physiological and anatomical characteristics of the urethra in female patients. Women have a short urethra, and the orifice of the urethra is close to the vagina and anus, which are easily contaminated.

This study had several limitations. First, these machine learning models were trained and developed based on single-center data. Single-center data may have the disadvantage of data bias and small sample size, which will affect the generalization ability and accuracy of the model. Second, all the operations we studied were unilateral and single-channel PCNL, so we were unable to investigate the influence of tract number on post-PCNL SIRS. Finally, we recognize that there may be differences in reference values between different laboratories, and the use of different measurement methods may lead to differences in results, which poses challenges to the accuracy and generalizability of using machine learning models to predict the occurrence of SIRS. The heterogeneity of different laboratory datas may affect the performance of the model, and special attention should be paid when applying the model to different laboratory settings [31]. In the future, we intend to carry out a multicenter study and collect data from multicenter to increase the sample size and improve the generalization ability of the model. In addition, we will conduct external validation, which can help identify and address the limitations of the model and improve its reliability and applicability.

## Conclusion

We identified relevant factors of post-PCNL SIRS and developed machine learning models to predict the possibility of SIRS after PCNL. We discovered that the SVM model has the potential to enhance data analysis and clinical decision-making capabilities.

## Data Availability

The datasets generated and/or analysed during the current study are not publicly available but are available from the corresponding author on reasonable request.
